# Comparison of Blood Concentration and Weight-Based Heparin and Protamine Dosing Strategies for Cardiopulmonary Bypass: A Systematic Review and Meta-Analysis

**DOI:** 10.7759/cureus.54144

**Published:** 2024-02-13

**Authors:** Gavin Raner, Yirui Hu, Cody Trowbridge, Li Zhang, John Logan, Xianren Wu, Xiaopeng Zhang

**Affiliations:** 1 Medicine and Surgery, School of Medicine, University of Western Ontario, London, CAN; 2 Population Health Sciences, Geisinger Commonwealth School of Medicine, Danville, USA; 3 Perfusionist, Geisinger Medical Center, Danville, USA; 4 Anesthesiology, Geisinger Medical Center, Danville, USA; 5 Anesthesiology, Johns Hopkins Bayview Medical Center, Danville, USA

**Keywords:** cardiopulmonary bypass, unfractionated heparin, big data analytics and machine learning, activated clotting time, systematic review and meta analysis, protamine sulfate, therapeutic anticoagulation

## Abstract

Background: The conventional method of heparin and protamine management during cardiopulmonary bypass (CPB) is based on total body weight which fails to account for the heterogeneous response to heparin in each patient. On the other hand, the literature is inconclusive on whether individualized anticoagulation management based on real-time blood heparin concentration improves post-CBP outcomes.

Methods: We searched databases of Medline, Excerpta Medica dataBASE (EMBASE), PubMed, Cumulative Index to Nursing and Allied Health Literature (CINHL), and Google Scholar, recruiting randomized controlled trials (RCTs) and prospective studies comparing the outcomes of dosing heparin and/or protamine based on measured heparin concentration versus patient's total body weight for CPB. Random effects meta-analyses and meta-regression were conducted to compare the outcome profiles. Primary endpoints include postoperative blood loss and the correlation with heparin and protamine doses, the reversal protamine and loading heparin dose ratio; secondary endpoints included postoperative platelet counts, antithrombin III, fibrinogen levels, activated prothrombin time (aPTT), incidences of heparin rebound, and re-exploration of chest wound for bleeding.

Results: Twenty-six studies, including 22 RCTs and four prospective cohort studies involving 3,810 patients, were included. Compared to body weight-based dosing, patients of individualized, heparin concentration-based group had significantly lower postoperative blood loss (mean difference (MD)=49.51 mL, 95% confidence interval (CI): 5.33-93.71), lower protamine-to-heparin dosing ratio (MD=-0.20, 95% CI: -0.32 ~ -0.12), and higher early postoperative platelet counts (MD=8.83, 95% CI: 2.07-15.59). The total heparin doses and protamine reversal were identified as predictors of postoperative blood loss by meta-regression.

Conclusions: There was a significant correlation between the doses of heparin and protamine with postoperative blood loss; therefore, précised dosing of both could be critical for reducing bleeding and transfusion requirements. Data from the enrolled studies indicated that compared to conventional weight-based dosing, individualized, blood concentration-based heparin and protamine dosing may have outcome benefits reducing postoperative blood loss. The dosing calculation of heparin based on the assumption of a one-compartment pharmacokinetic/pharmacodynamic (PK/PD) model and linear relationship between the calculated dose and blood heparin concentration may be inaccurate. With the recent advancement of the technologies of machine learning, individualized, precision management of anticoagulation for CPB may be possible in the near future.

## Introduction and background

Unfractionated heparin has been the most used anticoagulant for more than 70 years since the development of cardiopulmonary bypass (CPB). It is a heterogeneous mixture of negatively charged highly sulfated polysaccharides with molecular weights ranging between 5,000 and 30,000 kilo Dalton (kDa) [1]. The application of heparin is favored in CPB because of its predictable activity and reliable reversal with protamine. However, the ideal dosing of heparin and protamine remains controversial, partly because of the heterogeneity of patient responses. Furthermore, the available point-of-care (POC) activated clotting time (ACT) testing does not reliably correlate with the effect of heparin concentration [2]. Typically, heparin is administered as a bolus based on the patient’s total body weight, with a target ACT of 400-480 s considered safe for CPB, although this is not strictly evidence-based [3-5]. Several studies have suggested that a lower ACT is equivalent to preventing thrombosis during CPB [6,7]. However, to the best of our knowledge, no study has defined the lower or higher limits of ACT or heparin concentration. The thought that higher heparin doses may be relatively harmless could be clinically misleading [8].

The reversal dose of protamine was typically estimated based on the initial loading dose of heparin and additional doses of heparin administered at fixed intervals during CPB; clinical studies reported reversal protamine-to-heparin dose ratios ranging from 0.5 to 1.3. As these estimates are not based on real-time heparin concentrations, there is a risk of over- or underdosing protamine, which may result in hemodynamic instability, excessive bleeding from protamine-induced coagulopathy or insufficient reversal, and heparin rebound.

Individualized dosing of heparin and protamine based on measured heparin concentration may reduce complications caused by inappropriate heparin and/or protamine doses. Bull et al. introduced the concept of heparin dosing according to the measured heparin dose response [9,10]. A POC device (Hemostasis Management System, Medtronic) based on the same concept has been made available for more than three decades; however, published results regarding its outcome benefits have been mixed. Most studies have shown that the total heparin dose administered during CPB was significantly higher in the intervention groups; however, it remains unclear whether a higher dose of heparin increases postoperative bleeding. The heparin clearance half-life is dose dependent. Recent literature suggests that at higher doses, heparin metabolism may follow a two-compartment pharmacokinetic model [11]; therefore, the assumption of a linear relationship between the dose of heparin and the resulting ACT and/or heparin concentration may be misleading.

This systematic review and meta-analysis compared body weight-based versus blood heparin concentration-based anticoagulation management strategies for CPB. The primary endpoints included postoperative blood loss (reflected by chest tube output) and the correlation between heparin and protamine doses and postoperative bleeding. The secondary endpoints included the protamine and heparin dose ratio, heparin rebound, re-exploration of chest wound for bleeding, postoperative platelet counts, levels of antithrombin III, fibrinogen, and activated prothrombin time (aPTT).

## Review

Methods

Literature Search and Data Collection

We systematically searched Ovid Medline, Cochrane CENTRAL, PubMed, EMBASE, CINAHL, and Google Scholar for peer-reviewed full publications of randomized controlled trials (RCTs) or prospective cohort studies comparing acute postoperative outcomes between heparin concentration-based dosing and total body weight (TBW)-based dosing of heparin and protamine for CPB. The search terms used were heparin concentration, anticoagulation, protamine, ACT, CPB, and postoperative blood loss. We also manually searched for studies listed in the references of the enrolled articles. The search timeframe was between 1946 (the earliest year that publications were searchable online in Medline and EMBASE) and November 2023. There were no language limitations to this study. The PROSPERO registration number for this study is CRD42020172470.

Our inclusion criteria are as follows: (1) RCTs or prospective cohort studies published in peer-reviewed journals and (2) studies involving adult patients who underwent cardiac surgeries requiring CPB with heparin anticoagulation management based on total body weight or heparin concentration. Publications regarding pediatric patients and cardiac procedures without CPB were excluded.

The primary endpoints included postoperative blood loss and the correlation between heparin, protamine doses, and postoperative bleeding. The secondary endpoints included protamine-to-heparin dose ratio, incidence of heparin rebound, re-exploration of chest wound for bleeding, postoperative platelet counts, levels of antithrombin III, fibrinogen level, and aPTT.

The study selection was conducted in three screening steps. The first screening of articles identified from the literature search was independently reviewed by two reviewers (LZ and XZ), and discrepancies were resolved between the reviewers with the aid of a third reviewer (XW). In the second screening, full-text studies that met the inclusion criteria were included in the final review and meta-analysis. Data were collected from the selected studies by three reviewers (RG, LJ, and LZ) and were independently verified by two reviewers (XZ and XW). The characteristics of each study, including study design, patient baseline information, procedural details, and the abovementioned perioperative outcomes, were extracted into an Excel file.

Statistical Analyses

Meta-analyses were performed, using a random-effects model, to compare outcomes of blood heparin concentration- and body weight-based heparin and protamine management. Odds ratios (ORs) and 95% confidence intervals (CIs) were estimated for binary outcomes in eligible studies. The pooled ORs were considered statistically significant if the 95% CI did not span the number 1. The mean differences (MDs) and 95% CIs were estimated for continuous outcomes from the eligible studies. The pooled MDs were considered statistically significant if the 95% CI did not cover 0. Each study’s pooled estimates and measures of variability were used to generate forest plots. Publication bias was evaluated using Egger’s test. The variability among the included studies was assessed via heterogeneity tests using the I^2^ statistic. Meta-regression models were fitted between study-level covariates and the primary outcome of interest. p-values <0.05 were considered statistically significant. Statistical analyses were performed in R Studio (Version 1.0.136; The R Foundation, Vienna, Austria) using the “Meta” and “Metafor” packages [12] and Comprehensive Meta-analysis version 4 [13]. 

Results  

Qualitative Analysis 

**Figure 1:**
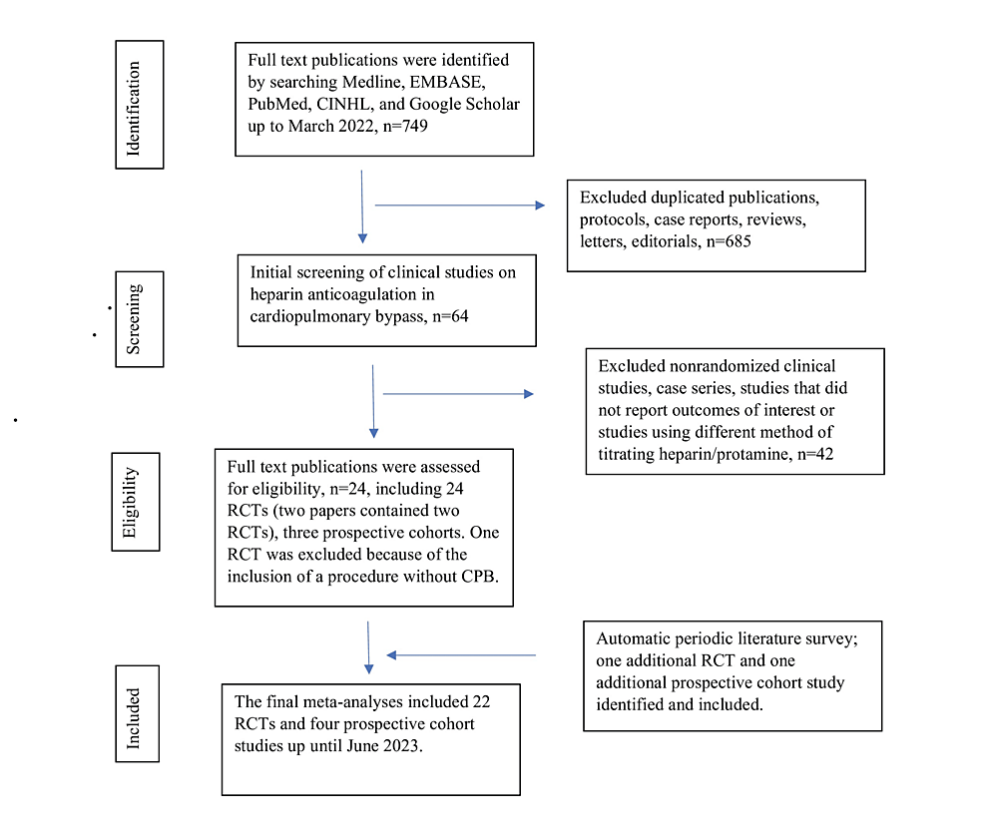
Flowchart of literature search and enrollment following the guideline of Preferred Reporting Items for Systematic Reviews and Meta-Analyses (PRISMA) CINHL: Cumulative Index to Nursing and Allied Health Literature, RCT: randomized controlled trial, CPB: cardiopulmonary bypass.

The database search yielded 749 citations. Of these, 685 studies were excluded because of duplication, irrelevant topics, or assessments of exposure or outcomes that did not meet the inclusion criteria. The 64 remaining articles were retrieved and examined in more detail. Twenty-two RCTs and four prospective cohort studies met the inclusion criteria and were included in this systematic review and meta-analysis (Figure 1).

Study Characteristics

There were 22 RCTs and four prospective studies involving 3,810 patients; the sample size of each study ranged from 12 to 254, with a median of 44 (Table 1). The Preferred Reporting Items for Systematic Reviews and Meta-Analyses (PRISMA) 2020 checklist is given in Appendix, Table 2.

**Table 1:** Characteristics of the trials enrolled in the meta-analysis RCT: randomized controlled trial, ECMO: extracorporeal membrane oxygenation, TxA: tranexamic acid, H & P: both heparin and protamine were dosed based on measured blood heparin concentration. H: heparin was dosed based on real-time blood heparin concentration, P: protamine reversal was dosed based on heparin concentration.

Study name	Country	Trial type	Journal	Study type	Sample size		Exclusion and reason	
					Intervention	Control	Intervention	Control
Gravlee et al. [14], 1990	USA	RCT	J Thorac Cardiovasc Surg	H & P	33	30	Re-exploration (2)	
Beholz et al. [15], 1999	Germany	RCT	Thorac Cardiovasc Surg	H & P	49	46	Re-exploration (5)	Re-exploration (2)
Despotis et al. [16], 1995	USA	RCT	J Thorac Cardiovasc Surg	H & P	127	127	Re-exploration (3)	Re-exploration (4)
Guarracino et al. [17], 2001	Hungary	RCT	Minerva Anestesiol	P	26	24	Heparin resistance (5)	
Hashimoto et al. [18], 1999	Japan	RCT	J Cardiovasc Surg	H & P	33	24	Unspecified (2)	
Hofmann et al. [19], 2013	Germany	RCT	Perfusion	H & P	29	24	Re-exploration, incomplete lab data (7)	
Kjellberg et al. [20], 2019	Sweden	RCT	J Cardiothorac Vasc Anesth	H & P	19	20	ECMO (2)	
Koster et al. [21], 2002	Germany	RCT	Anesthesiology	H & P	100	100		
Koster et al. [22], 2014	Germany	RCT	Clin Appl Thromb/Hemost	P	15	15		
Miles et al. [23], 2021	UK	Prospective cohort	PLoS Med	P	30	30		
Noui et al. [24], 2012	France	Prospective cohort	Perfusion	H & P	22	22		
Ohata et al. [25], 1999	Japan	RCT	Jpn J Thorac Cardiovasc Surg	P	12	8		
Pappalardo et al. [26], 2006	Italy	RCT	Perfusion	H & P	17	22		
Radulovic et al. [27], 2015	Sweeden	RCT	PLOS One	H & P	33	31	Change of surgery (3)	Withdraw informed consent (1)
Runge et al. [28], 2009	Denmark	Prospective cohort	JECT	H & P	28	25		
Shigeta et al. [29], 1999	Japan	RCT	J Thorac Cardiovasc Surg	H & P	20	14	Received platelet (2)	Unspecified (6)
Shirota et al. [30], 2000	Japan	RCT	Artif Organs	H	5	5		
Sakurada et al. [31], 1997	Japan	RCT	Nippon Kyobu Geka Gakkai Zasshi	H & P	19	15		
Shore-Lesserson et al. [32], 1998	USA	RCT	Can J Anaesth		36 (H), 18 (H), 28 (P)	53	Re-exploration (3), Received TxA(4), wrong heparin dose (1)	
Slight et al. [33], 2008	UK	RCT	J Cardiothorac Vasc Anesth	H & P	18	20		
Vonk et al. [34], 2014	Netherlands	RCT	J Cardiothorac Vasc Anesth	H & P	19	19	Change of surgery (6)	
Jobes et al. [35], 1995	USA	RCT	J Thorac Cardiovasc Surg	H & P	22	24	Wrong heparin dose (3)	Re-exploration (1), wrong heparin dose (1)
Yamanishi et al. [36], 1997	Japan	RCT	Kyobu Geka	H & P	21	11		
Bailly et al. [37], 2021	France	Prospective cohort	Minerva Anestesiol	H & P	96	92		
Li et al. [38], 2022	Canada	RCT	Can J Anaesth	H & P	50	50		
Nuttall et al. [39], 2022	USA	RCT	Ann Thorac Cardiovasc Surg	H 7 P	91	90		

Quality Assessments

**Figure 2:**
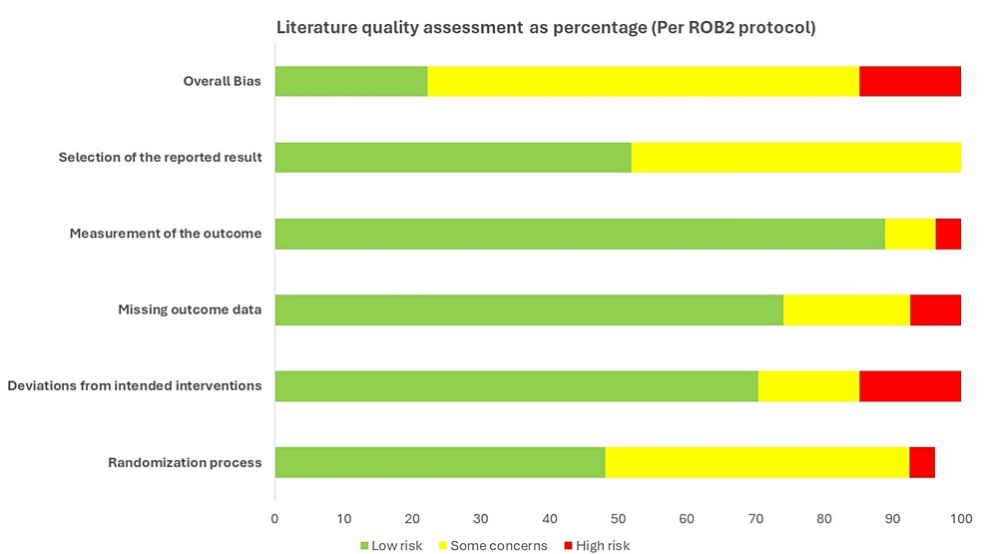
The quality assessment of the enrolled studies using RoB 2 risk-of-bias tool. RoB 2: risk of bias 2.

The quality of the enrolled trials was scored using risk of bias 2 (RoB 2) [40] as a risk-of-bias tool for randomization. RoB 2 is structured into a fixed set of bias domains, focusing on different aspects of trial design, conduct, and reporting. Within each domain, a series of signaling questions aim to elicit information about the features of the trial that are relevant to the risk of bias. Judgments can be categorized as having a “low” or “high” risk of bias. The overall quality of the evaluation indicated that 85.2% of the studies had low risk and some concern of the bias, and 14.8% had high risk (Figure 2).

Quantitative Analysis

**Figure 3:**
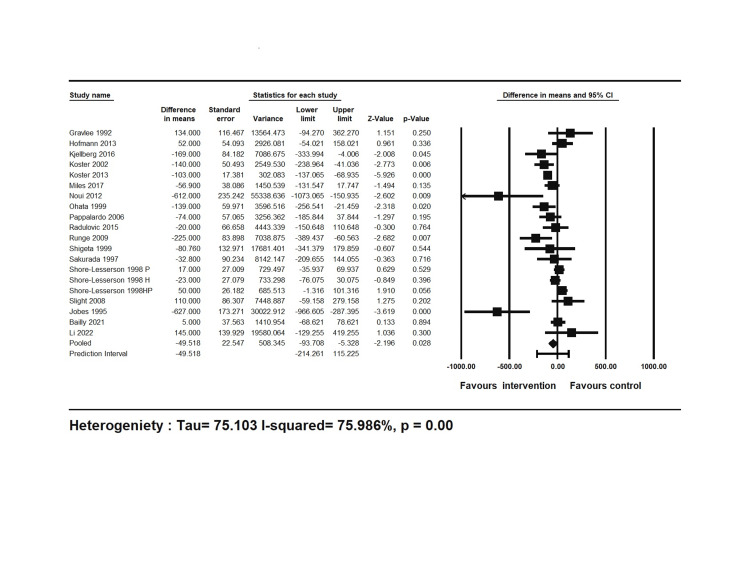
Forest plot of the postoperative cumulative chest tube output in the intervention and control groups CI: confidence interval.

Postoperative blood loss and the correlation of protamine dose and total heparin dose are as follows: 22 studies reported chest tube output, an indicator of blood loss, up to 24 hours post-surgery, of which 19 reported it as mean and standard deviation (SD) or median with interquartile ranges (IQRs). The random-effects meta-analysis indicated that heparin concentration (HC)-based management resulted in significantly lower postoperative blood loss compared to the control group (MD=49.52 mL, 95% CI: 5.33-93.71 mL) (Figure 3).

**Figure 4:**
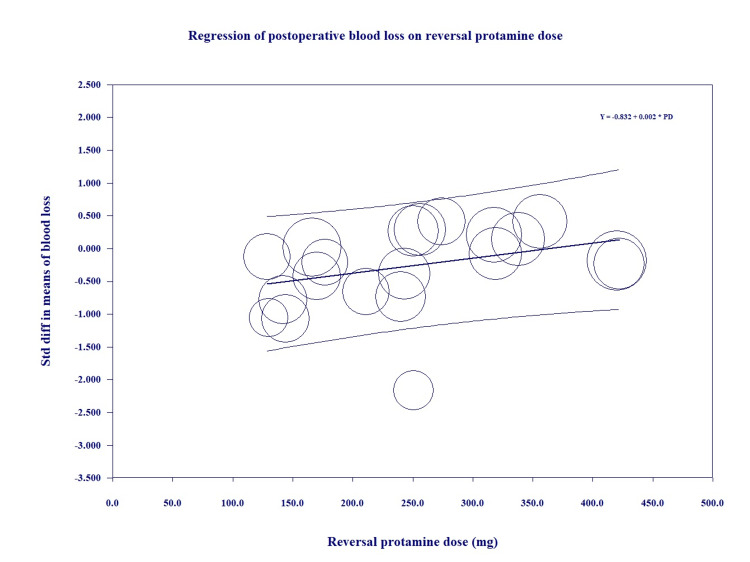
Meta-regression of blood loss on reversal dose of protamine Y: standard difference in means of blood loss, PD: protamine dose.

**Figure 5:**
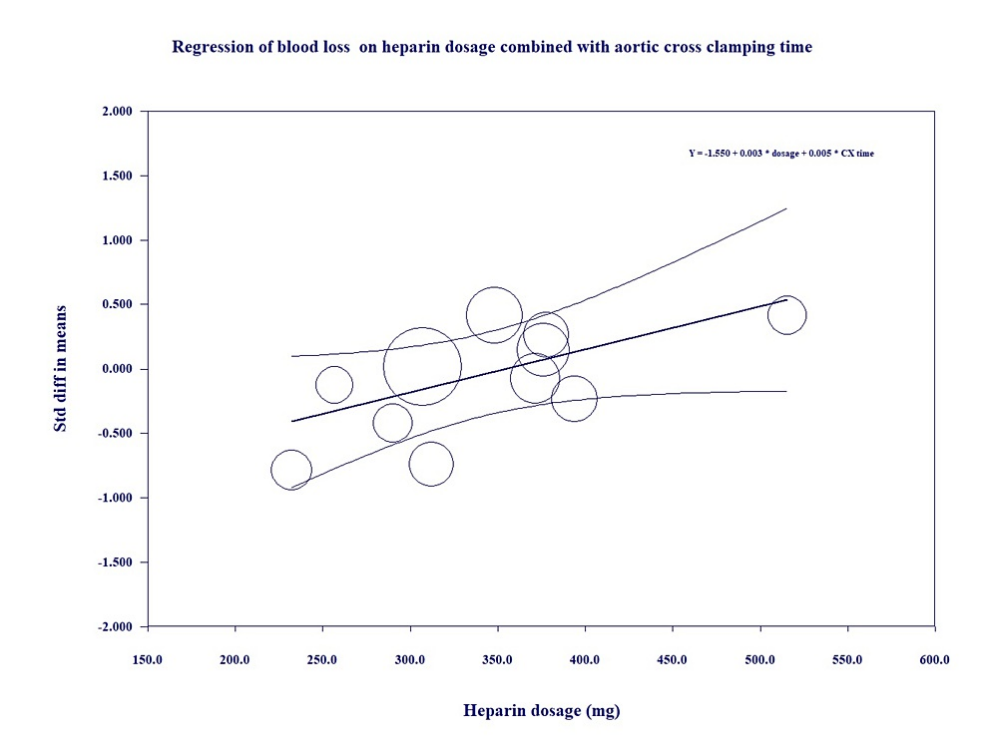
Meta-regression of blood loss on combined heparin dose with aortic cross-clamping times Y: standard difference in means of blood loss; CX: aortic cross-clamping time.

We further conducted a random-effects meta-regression analysis for total heparin dose, protamine dose and protamine/heparin dose ratio, duration of CPB, and duration of aortic cross-clamping on the MDs regarding the postoperative blood loss between the intervention and control groups. Protamine dose was an independent predictor of postoperative blood loss (coefficient=0.0023, p=0.05) (Figure 4); total heparin dose was associated with postoperative blood loss between the groups with long aortic cross-clamping time (coefficient=0.0033, p=0.01) (Figure 5). The relationship between total heparin doses and the outcomes remained consistent when additional study-level covariates were included in the meta-regression.

Heparin, Protamine Dosage, and the Protamine/Heparin Dose Ratio

**Figure 6:**
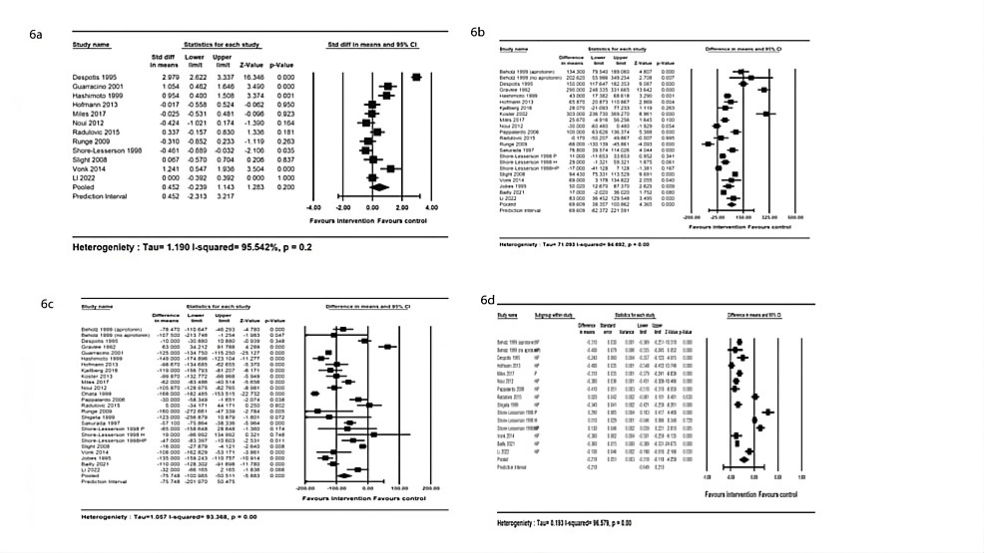
Forest plot of the initial loading dose, cumulative total heparin dose, reversal protamine dose, and reversal protamine-to-heparin dose ratio 6a, There was no significant difference in the initial loading dose of heparin between intervention and control groups. 6b, The cumulative total dose of heparin used during the CPB was significantly higher in the intervention group. 6c, The protamine reversal dose was significantly lower in the intervention. 6d, The reversal protamine-to-heparin dose ratio was significantly lower in the intervention compared to the control groups. CPB: cardiopulmonary bypass.

Eighteen studies compared the total heparin doses and the ratios of reversal protamine-to-heparin dose in the intervention and control groups. The random-effects meta-analysis indicated that although there were no significant differences in initial heparin loading dose between the groups (Figure 6a), the intervention group had significantly higher total heparin doses (MD=69.61 mg, 95% CI: 38.36-100.87 mg) (Figure 6b), lower protamine dosage (MD=-75.75 mg, 95% CI: -100.99 to -50.52 mg) (Figure 6c), and lower ratio between protamine reversal and total heparin dose (MD=-0.20, 95% CI: -0.12 to - 0.32) (Figure 6d). Compared to the body weight-based group, Egger’s test indicated no potential publication bias for heparin (p=0.76) or protamine dosage (p=0.72) in the included studies.

 Post-CPB Platelet Counts, Antithrombin III, and aPTT

**Figure 7:**
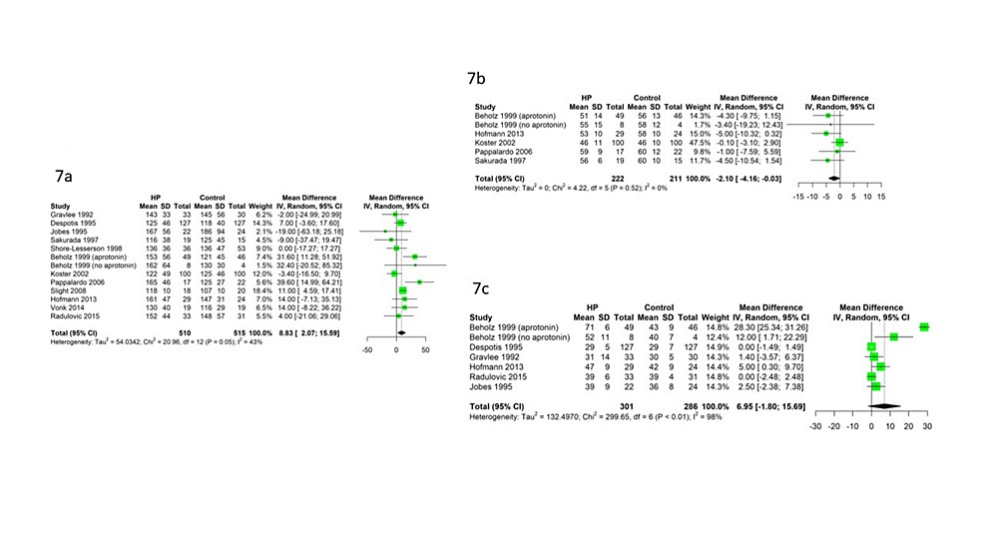
Forest plot of postoperative platelet counts, postoperative antithrombin III level (in percentage), and aPTT level 7a, The platelet count was significantly higher in the intervention compared to the control. 7b, The antithrombin level was significantly lower in the intervention group.  7c, There was no significant difference in postoperative aPTT levels between the groups. aPTT: activated prothrombin time.

Thirteen studies reported post-CPB platelet counts early in the intensive care unit. The random-effects meta-analysis indicated that the intervention groups had significantly higher post-CPB platelet counts compared to the control groups (MD=8.83, 95% CI: 2.07-15.59) with heterogeneity (I^2^=43%) (Figure 7a). Six studies reported postoperative antithrombin III and seven studies compared the aPTT. The random-effects meta-analysis indicated that the intervention group had significantly lower post-CPB antithrombin III than the control group (MD=-2.10%, 95% CI: -4.16% to 0.03%) without heterogeneity (I^2^=0%) (Figure 7b); the small effect size difference (mean of 2.1%) may not indicate clinical significance. There were no significant differences in postoperative fibrinogen and aPTT levels between the groups (Figure 7c).

Heparin Rebound and Re-Exploration of the Chest Wound Due to Bleeding

Only six studies reported heparin rebound, and seven reported reopening due to bleeding. Random-effects meta-analysis indicated that, compared to TBW dosing, the heparin concentration-based management is not associated with increased heparin rebound (OR=1.25, 95% CI: 0.68-2.30, I^2^=56%) and reopening due to bleeding (OR=0.82, 95% CI: 0.43-1.59, I^2^=0%).   

Discussion

The data from the current meta-analysis indicate that individualized anticoagulation management based on measured blood heparin concentration may reduce postoperative bleeding through the preservation of postoperative platelet counts, reduced protamine dose for reversal, as well as the reduction of reversal protamine to total heparin dose ratio. The results from the meta-regression suggested that the protamine overdose was significantly correlated with increasing postoperative bleeding. In addition, there was a significant correlation between increased total heparin dose along with prolonged aortic clamping time and postoperative bleeding. These data suggest that an overdose of both heparin and protamine may have a negative impact on postoperative blood loss, possibly due to reduced platelet counts and function.

There may be a confounding issue of inaccurate calculation of heparin loading dose because, noticeably, the data revealed that there was no significant difference in initial heparin loading doses between the groups; however, the total heparin dose was significantly higher in the intervention group, and the antithrombin III was significantly lower. These results suggest that higher additional doses of heparin were administered to the intervention group during CPB.

The meta-regression data indicated that an increased total heparin dose with a prolonged aortic cross-clamping time was significantly correlated with postoperative blood loss, suggesting that more heparin was required if the pump run was longer. In addition, there was a possibility of miscalculation of heparin doses. In the enrolled RCTs and prospective studies, the method of heparin loading dose calculation was based on the assumption of heparin metabolism following a one-compartment pharmacokinetic/pharmacodynamic (PK/PD) model; therefore, the heparin dose-response curve must be linear [9]. However, recent studies have shown that at high doses, such as when initiating CPB, a two-compartment heparin PK/PD model may be more accurate and that the relationship between the heparin dose and ACT or blood heparin concentration may likely be nonlinear [11,41]. The fact that the half-life of heparin clearance is dose-dependent suggests that heparin does not simply follow a one-compartment PK/PD model; therefore, the calculated heparin loading dose may be misleading. We believe that accurate heparin dose calculation may require new technologies, such as machine learning, which offer greater data processing power to generate accurate mathematical equations.

The intervention groups demonstrated a consistently lower protamine dose requirement for heparin reversal and a significantly lower protamine-to-heparin dose ratio (0.68-0.88:1). Current guidelines recommend a ratio of up to 1:1 for protamine-to-heparin loading dose after CPB [42]. However, data from recent literature indicate that this ratio may be too high and associated with increased postoperative bleeding and blood transfusions [43]. Some studies have revealed that a protamine-to-heparin dose ratio as low as 0.5:1 was sufficient to neutralize residual heparin following CPB [44-46].

Protamine and heparin overdoses were associated with significantly reduced platelet counts and dysfunction [47-49]. Therefore, our recommendations regarding protamine dosing are threefold. First, protamine titration based on individualized heparin dose response and real-time measurement of blood heparin concentration could be a safer alternative to conventional body weight-based methods. Second, a reduced target protamine-to-heparin dose ratio can help maintain adequate hemostasis and minimize the need for blood transfusions. Finally, because heparin may follow a two-compartment PK/PD model-based distribution and clearance, additional postoperative application of protamine may be beneficial [11].

Our study indicated that the requirements for heparin and protamine dosages may be reduced for managing anticoagulation for CPB. The 2018 Society for Thoracic Surgeon (STS)/Society of Cardiovascular Anesthesiologists (SCA)/American Society of ExtraCorporeal Technology (AmSECT) Clinical Practice Guidelines recommended a post-heparin target ACT of 400-480 s which was not based on clinical trials [3,14]. However, evidence suggests that targeting lower ACTs with a lower heparin dose may be sufficient to prevent thrombosis during CPB [50,51]. A blood heparin concentration of 4.0 IU/mL was considered safe to initiate CPB, although levels as low as 2.7 IU/ml were acceptable [52]. Although blood heparin concentration would provide the most accurate monitoring of the patient’s heparin response, its application could be unintuitive. Clinical ACT, not the real-time measurement of blood heparin concentration, remains the conventional monitoring method because it is a POC test easily performed in the operating theater; therefore, some enrolled trials applied ACT to replace heparin concentration without considering the fact that ACT, under certain clinical conditions, may not correlate well with blood heparin concentration, especially during CPB, when the patient is under conditions of hypothermia and hemodilution [7,53-56]. We suspect this could be a significant confounding factor for the heparin dosing management. it was known that monitoring the heparin dose response through a combination of ACT, blood heparin concentration, and other coagulation tests, such as rotational thromboelastometry (ROTEM), thromboelastography (TEG), and factor Xa assays, could provide a comprehensive guide for managing anticoagulation during CPB. However, this is beyond the scope of the current study.

This study had several important limitations. First, the enrolled RCTs were published at various times over the past 30 years. Over this somewhat lengthy period, there have been dramatic improvements in the fields of anesthesia and cardiac surgery. One such change is the introduction of heparin-coated circuits, which are now the standard in CPB. However, the guidelines for managing heparin anticoagulation have not changed. Although the question of adequate ACT or heparin concentration for CPB was beyond the scope of this study, we believe that this could be a significant confounding issue due to the non-correlation of ACT and heparin concentration under certain circumstances. Second, owing to technological advancements, an increasing number of quantifying coagulation tests are now available as POC tests, such as ROTEM or TEG, the combination of which could provide accurate guidance for heparin management. However, this was not observed in any of the published RCTs. Therefore, in this systematic review and meta-analysis, data from these quantifiable assays could not be synthesized and presented because of their scarcity. Third, some of the included RCTs, although conducted well from a clinical perspective, were underpowered because of their small sample sizes. Lastly, while ACT remains the dominant monitoring method for heparin anticoagulation due to its user-friendliness, it is well known that using ACT as a surrogate for measured heparin concentration may be misleading due to poor correlation during CPB. Literature suggested that, although not a POC test, anti-Xa assay may applied in conjunction with the ACT to effectively monitor the adequacy of anticoagulation and predict perioperative bleeding [57].

## Conclusions

There was a significant correlation between the doses of heparin and protamine with postoperative blood loss; therefore, précised dosing of both could be critical for reducing bleeding and transfusion requirements. Data from the enrolled studies indicated that compared to conventional weight-based dosing, individualized, blood concentration-based heparin and protamine dosing may have outcome benefits reducing postoperative blood loss. The dosing calculation of heparin based on the assumption of a one-compartment PK/PD model and linear relationship between the calculated dose and blood heparin concentration may be inaccurate. With the recent advancement of the technologies of machine learning, individualized, precision management of anticoagulation for CPB may be possible in the near future.

## Appendices

**Table 2:** PRISMA 2020 checklist PRISMA: Preferred Reporting Items for Systematic Reviews and Meta-Analyses.

Section and topic	Item #	Checklist item	Location where item is reported
TITLE			
Title	1	Identify the report as a systematic review.	Pg 1
ABSTRACT			
Abstract	2	See the PRISMA 2020 for Abstracts checklist.	Pg 2, 3
INTRODUCTION			
Rationale	3	Describe the rationale for the review in the context of existing knowledge.	Pg 4
Objectives	4	Provide an explicit statement of the objective(s) or question(s) the review addresses.	Pg 5
METHODS			
Eligibility criteria	5	Specify the inclusion and exclusion criteria for the review and how studies were grouped for the syntheses.	Pg 5, 6
Information sources	6	Specify all databases, registers, websites, organizations, reference lists, and other sources searched or consulted to identify studies. Specify the date when each source was last searched or consulted.	Pg 5
Search strategy	7	Present the full search strategies for all databases, registers, and websites, including any filters and limits used.	Pg 5
Selection process	8	Specify the methods used to decide whether a study met the inclusion criteria of the review, including how many reviewers screened each record and each report retrieved, whether they worked independently, and if applicable, details of automation tools used in the process.	Pg 6
Data collection process	9	Specify the methods used to collect data from reports, including how many reviewers collected data from each report, whether they worked independently, any processes for obtaining or confirming data from study investigators, and if applicable, details of automation tools used in the process.	Pg 6
Data items	10a	List and define all outcomes for which data were sought. Specify whether all results that were compatible with each outcome domain in each study were sought (e.g., for all measures, time points, analyses), and if not, the methods used to decide which results to collect.	Pg 6
	10b	List and define all other variables for which data were sought (e.g., participant and intervention characteristics, funding sources). Describe any assumptions made about any missing or unclear information.	Pg 6
Study risk of bias assessment	11	Specify the methods used to assess the risk of bias in the included studies, including details of the tool(s) used, how many reviewers assessed each study and whether they worked independently, and if applicable, details of automation tools used in the process.	Pg 5, 6
Effect measures	12	Specify for each outcome the effect measure(s) (e.g., risk ratio, mean difference) used in the synthesis or presentation of results.	Pg 6
Synthesis methods	13a	Describe the processes used to decide which studies were eligible for each synthesis (e.g., tabulating the study intervention characteristics and comparing against the planned groups for each synthesis (item #5)).	Pg 5, 6
	13b	Describe any methods required to prepare the data for presentation or synthesis, such as handling of missing summary statistics, or data conversions.	Pg 7, 8
	13c	Describe any methods used to tabulate or visually display the results of individual studies and syntheses.	Pg 8-11
	13d	Describe any methods used to synthesize results and provide a rationale for the choice(s). If meta-analysis was performed, describe the model(s), method(s) to identify the presence and extent of statistical heterogeneity, and software package(s) used.	Pg 6
	13e	Describe any methods used to explore possible causes of heterogeneity among study results (e.g., subgroup analysis, meta-regression).	Pg 6
	13f	Describe any sensitivity analyses conducted to assess the robustness of the synthesized results.	
Reporting bias assessment	14	Describe any methods used to assess the risk of bias due to missing results in a synthesis (arising from reporting biases).	Pg 6
Certainty assessment	15	Describe any methods used to assess certainty (or confidence) in the body of evidence for an outcome.	Pg 6
RESULTS			
Study selection	16a	Describe the results of the search and selection process, from the number of records identified in the search to the number of studies included in the review, ideally using a flow diagram.	Pg 7
	16b	Cite studies that might appear to meet the inclusion criteria, but which were excluded, and explain why they were excluded.	Pg 7
Study characteristics	17	Cite each included study and present its characteristics.	Pg 8-10
Risk of bias in studies	18	Present assessments of risk of bias for each included study.	Pg 11, appendix
Results of individual studies	19	For all outcomes, present, for each study: (a) summary statistics for each group (where appropriate) and (b) an effect estimate and its precision (e.g., confidence/credible interval), ideally using structured tables or plots.	Pg 11-16
Results of syntheses	20a	For each synthesis, briefly summarize the characteristics and risk of bias among contributing studies.	Pg 11-16
	20b	Present results of all statistical syntheses conducted. If meta-analysis was done, present for each the summary estimate and its precision (e.g., confidence/credible interval) and measures of statistical heterogeneity. If comparing groups, describe the direction of the effect.	Pg 11-16
	20c	Present results of all investigations of possible causes of heterogeneity among study results.	Pg 12-20
	20d	Present results of all sensitivity analyses conducted to assess the robustness of the synthesized results.	Pg 12-20
Reporting biases	21	Present assessments of risk of bias due to missing results (arising from reporting biases) for each synthesis assessed.	Pg 12-20
Certainty of evidence	22	Present assessments of certainty (or confidence) in the body of evidence for each outcome assessed.	Pg 11-16
DISCUSSION			
Discussion	23a	Provide a general interpretation of the results in the context of other evidence.	Pg 16-19
	23b	Discuss any limitations of the evidence included in the review.	Pg 18-19
	23c	Discuss any limitations of the review processes used.	Pg 18-19
	23d	Discuss the implications of the results for practice, policy, and future research.	Pg 19
OTHER INFORMATION			
Registration and protocol	24a	Provide registration information for the review, including register name and registration number, or state that the review was not registered.	Pg 6
	24b	Indicate where the review protocol can be accessed, or state that a protocol was not prepared.	Appendix 1
	24c	Describe and explain any amendments to information provided at registration or in the protocol.	n/a
Support	25	Describe sources of financial or non-financial support for the review, and the role of the funders or sponsors in the review.	Pg 19
Competing interests	26	Declare any competing interests of review authors.	Pg 19
Availability of data, code, and other materials	27	Report which of the following are publicly available and where they can be found: template data collection forms; data extracted from included studies; data used for all analyses; analytic code; and any other materials used in the review.	
